# Germline Mutations Including the Rare Pathogenic Variant c.3206delC in the *ATM* Gene Cause Ataxia Teleangiectasia-Associated Primary Central Nervous System Lymphoma

**DOI:** 10.3390/children8060469

**Published:** 2021-06-02

**Authors:** Jan R. Dörr, Anne Thorwarth, Agnieszka Mizia-Malarz, Josefine Radke, Anna Tietze, Pablo Hernáiz-Driever, Denise Horn, Alexander Gratopp, Angelika Eggert, Hedwig E. Deubzer

**Affiliations:** 1 Department of Pediatric Hematology and Oncology, Charité–Universitätsmedizin Berlin, 13353 Berlin, Germany; jan-rafael.doerr@charite.de (J.R.D.); anne.thorwarth@charite.de (A.T.); pablo.hernaiz@charite.de (P.H.-D.); angelika.eggert@charite.de (A.E.); 2 Berliner Institut für Gesundheitsforschung (BIH), 10178 Berlin, Germany; josefine.radke@charite.de; 3 Department of Pediatric Oncology, Hematology and Chemotherapy, Upper Silesia Children’s Care Health Center, Medical University of Silesia, 40-752 Katowice, Poland; amizia-malarz@sum.edu.pl; 4 Department of Neuropathology, Charité–Universitätsmedizin Berlin, 10117 Berlin, Germany; 5 German Cancer Consortium (Deutsches Konsortium für Translationale Krebsforschung; DKTK), Partner Site Berlin, 10115 Berlin, Germany; 6 German Cancer Research Center Heidelberg (Deutsches Krebsforschungszentrum; DKFZ), 69120 Heidelberg, Germany; 7 Department of Neuroradiology, Charité–Universitätsmedizin Berlin, 13353 Berlin, Germany; anna.tietze@charite.de; 8 Institute of Medical Genetics and Human Genetics, Charité–Universitätsmedizin Berlin, 13353 Berlin, Germany; denise.horn@charite.de; 9 Department of Pediatric Respiratory Medicine, Immunology and Critical Care Medicine, Charité–Universitätsmedizin Berlin, 13353 Berlin, Germany; 10 Experimental and Clinical Research Center (ECRC) of the Charité–Universitätsmedizin Berlin and the Max-Delbrück-Center for Molecular Medicine (MDC) in the Helmholtz Association, 13125 Berlin, Germany

**Keywords:** cancer, cancer predisposition, immune deficiency disorder, multiresistant bacteria, chemotherapy tolerance

## Abstract

We here report the case of a 2-year-old patient with a primary central nervous system lymphoma of B-cell origin. Due to their past medical history of repeated respiratory tract infections and the marked chemotherapy-associated toxicity and infectious comorbidity, we suspected that the patient also suffered from an inherited immune deficiency disorder. Despite the lack of classical pathognomonic symptoms for ataxia teleangiectasia and missing evidence for a cancer predisposition syndrome in the family, genetic testing identified biallelic germline mutations, including the rare pathogenic variant c.3206delC (p.Pro1069Leufs*2), in the ataxia telangiectasia-mutated (*ATM*) gene. The case highlights the importance of searching for immune deficiency disorders associated with primary central nervous system lymphoma before treatment initiation and the urgent need to develop novel treatment strategies for cancer patients with underlying immunodeficiency syndromes.

## 1. Introduction

Primary central nervous system lymphomas (PCNSL) are malignant, extranodal non-Hodgkin lymphomas (NHL) of the brain, the eye or the leptomeninges [1]. While PCNSL account for approximately 3–5% of all brain tumors in immunocompetent patients and around 5% of all NHL in adults, they are extremely rare in children. Overall, around 100 pediatric cases of PCNSL have been published so far [2]. In total, seven children with PCNSL were treated at the Department of Paediatric Oncology at the Charité–Universitätsmedizin Berlin in the last 35 years [3].

The large majority of PCNSL are diffuse large B-cell lymphomas [4]. Burkitt lymphomas, anaplastic large cell lymphomas or T-cell lymphomas have been rarely diagnosed [4]. PCNSL are histologically more homogenous than aggressive lymphomas that manifest in other localizations. They show a vasocentric growth pattern and high proliferation, which facilitates their infiltration of the central nervous system [5]. In adult patients with PCNSL, molecular analyses have mostly identified an activated B-cell-like subtype with mutations in genes, which mainly affect the JAK-STAT, NFkB and B-cell receptor signaling pathways—for example, *MYD88* and *CD79B*—as well as overexpression of *MYC*, *BCL2* and *BCL6* [1]. A similar genetic analysis of pediatric PCNSL has not yet been performed.

Immunocompromised patients have an increased risk of developing a PNCSL. In adults, the immunodeficiency is mostly acquired through chronic infections mediated through the Epstein–Barr virus, triggering the malignant transformation of B cells [6], or the human immunodeficiency virus, causing the acquired immunodeficiency syndrome AIDS. Additionally, immunosuppressive regimens after organ transplantation increase the risk of developing a PCNSL. In children, the development of PCNSL is frequently associated with inherited immunodeficiency syndromes, for example the severe congenital immune deficiency syndrome, the X-linked lymphoproliferative syndrome, the Wiskott Aldrich syndrome or ataxia telangiectasia [7].

Several parameters, such as age at diagnosis, histopathological subtype and immune status, determine the survival probability of patients with PCNSL. While the 5-year event-free survival rate of adult PCNSL patients only ranges between 10 and 20% after completion of a combined chemo- and radiation therapy-based regimen, immunocompetent children with a PCNSL have a more favorable prognosis, with a 5-year event-free survival rate of 70% [8]. However, children suffering from a PCNSL linked to an immunodeficiency syndrome show a markedly reduced survival probability [9]. We here report the clinical course of a 2-year-old Caucasian patient with a highly aggressive B-cell PSCNL on the basis of a so far undiagnosed ataxia teleangiectasia, which was characterized by considerably reduced chemotherapy tolerance and severe infectious complications mediated by multiresistant bacteria following multiple antibiotic treatment episodes due to respiratory tract infections since early infancy.

## 2. Case Presentation

**Figure 1:**
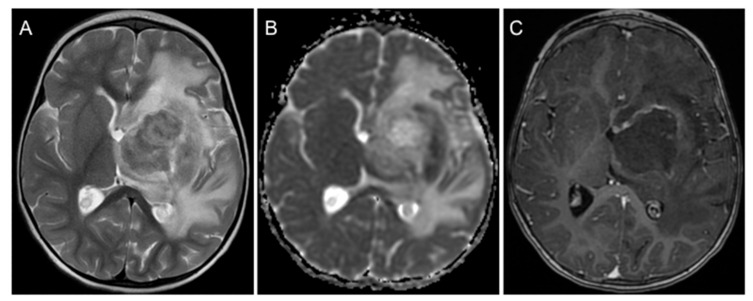
Imaging features of primary central nervous system lymphoma. (**A**) An axial T2-weighted magnetic resonance image shows a large left-sided mass primarily located in the basal ganglia/thalamus with considerable perifocal edema. (**B**) Diffusion-weighted imaging (apparent diffusion coefficient map) demonstrates diffusion restriction in the periphery of the tumor, indicating high cellularity. (**C**) Following intravenous contrast medium administration, relatively little inhomogeneous enhancement is seen at the margins of the mass.

The 2-year-old patient presented in reduced overall condition in our emergency department. She suffered from fever, partial respiratory failure, impaired consciousness with a Glasgow Coma Scale of 11, hemiparesis of the right arm, ptosis of the left eye and an intermittently insufficient swallowing reflex. The patient had previously been diagnosed with a PCNSL at the Medical University of Silesia in Katowice, Poland. The lymphoma was histopathologically classified as a diffuse large B-cell lymphoma. Initial imaging studies of the brain displayed a large, space-occupying mass centered in the left basal ganglia and the thalamus, inducing a midline shift and enlargement of the right ventricle due to obstruction of the foramen of Monro (Figure 1).

**Figure 2:**
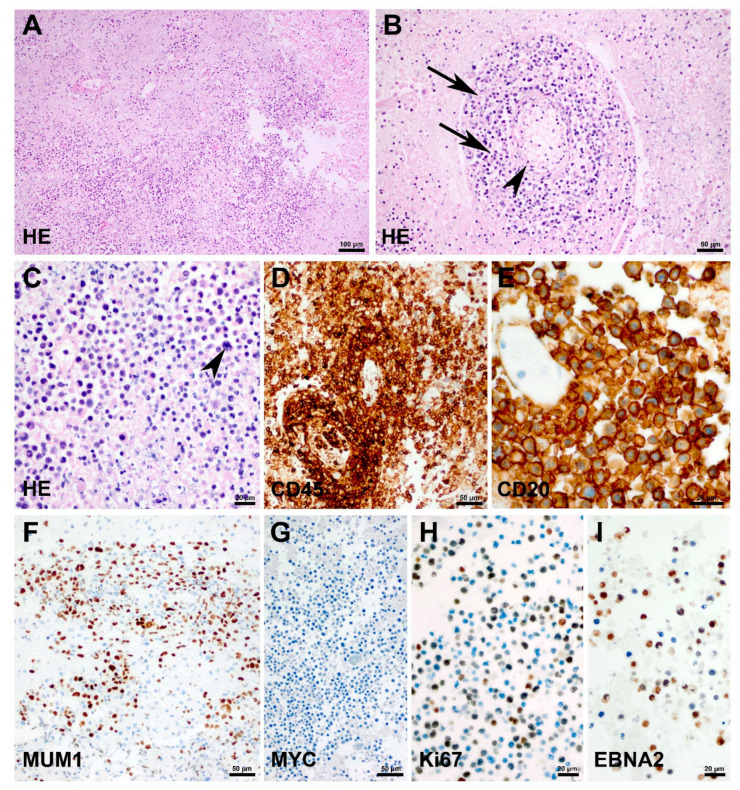
Histopathology of primary central nervous system lymphoma. Hematoxylin and eosin staining of the brain biopsy specimen (**A**–**C**) shows dense infiltrates of malignant lymphocytes with a characteristic accumulation of tumor cells in the perivascular space (**B**, arrows) of small intraparenchymal vessels (**B**; arrowhead). (**C**) The tumor is composed of blasts with large nuclei, prominent nucleoli and frequent mitotic figures (arrowhead). (**D**) Expression of CD45 on malignant B cells. (**E**) Expression of CD20 on malignant B cells. (**F**) Expression of Mum1 on malignant B cells. (**G**) Lack of MYC expression on lymphoma cells. (**H**) High proliferative activity as demonstrated by Ki67/MIB-1 labelling. (**I**) Positive EBNA2 expression as a sign for Epstein–Barr virus infected lymphoma cells.

Following partial neurosurgical resection and implantation of a ventriculoperitoneal shunt, the patient had received two chemotherapy cycles, i.e., COP and R-COP (rituximab, cyclophosphamide, vincristine and prednisolone), in line with the Inter B NHL 2010 protocol (National Clinical Trial Number: NCT01516580, European Clinical Trials (EudraCT) Number: 2010-019224-31, Date of Ethics Committee Approval: 6 June 2012) before presenting at our hospital for treatment continuation. At admission to our hospital, laboratory findings included a central hypothyroidism, a central adrenal insufficiency as well as a diabetes insipidus, altogether resulting from a PCNSL-induced impairment of the pituitary gland. Furthermore, the infectious disease parameters were strongly elevated. Microbiological examination of the tracheal secretions and blood cultures demonstrated a multiresistant *Pseudomonas aeruginosa* sepsis. Altogether, the patient required immediate and prolonged intensive care measurements, including mechanical ventilation. The past medical history of the patient was characterized by partly severe upper and lower respiratory tract infections requiring repeated antibiotic therapy since early infancy. The in-house histopathological examination confirmed the diagnosis of a diffuse large B-cell lymphoma and detected the Epstein–Barr virus and Epstein–Barr virus latent antigens such as EBNA2 in the B lymphoma cells (Figure 2).

The past medical history of repeated infections, the extensive volume of the PCNSL and the persistence of Epstein–Barr virus in the lymphoma cells prompted us to screen for germline mutations associated with an underlying immunodeficiency syndrome. Despite the absence of pathognomonic symptoms such as telangiectasias on the skin and the conjunctiva or the onset of neurological symptoms such as gait instability, we suspected that the patient may suffer from ataxia teleangiectasia, also known as Louis–Bar syndrome. It is an autosomal recessive disorder with mutations in the gene encoding the ataxia teleangiectasia-mutated (ATM) protein that is characterized by progressive neurodegeneration and a significantly increased susceptibility to malignancies such as leukemias and lymphomas [10]. Additionally, patients frequently suffer from recurrent infections and increased radiation sensitivity [10]. The *ATM* gene encodes a serine/threonine kinase, which plays a major role in controlling cell cycle checkpoint signaling pathways that are required for cellular responses to DNA damage and for genome stability. Indeed, hybridization capture followed by both high-throughput sequencing on an Illumina sequencing platform and Sanger sequencing identified two different mutations in the *ATM* gene in peripheral blood leukocytes from the patient. Firstly, we detected the point mutation c.7630-2A>C (splice acceptor), which results in an aberrant splicing of the gene transcript and consequently in a complete loss of gene function [11]. This mutation has previously been described as a germline mutation, which raises the susceptibility to different cancers—for example, breast cancer, pancreatic carcinoma, prostate carcinoma and lung carcinoma [12,13,14,15]. Secondly, we identified the rare frameshift mutation c.3206delC (p.Pro1069Leufs*2), which has previously been reported in a 4-year-old child that underwent pre-emptive allogeneic hematopoietic stem cell transplantation to correct the immunodeficiency and reduce the high risk for developing a hematological malignancy [16]. This mutation alters the reading frame and creates a premature stop codon, which likely triggers a nonsense-mediated mRNA decay. In order to investigate the genetic constellation of the identified mutations in the *ATM* gene of the patient’s germline, we tested peripheral blood leukocytes from both parents for the presence of *ATM* mutations by polymerase chain reaction (PCR)-based *ATM* gene sequencing. These analyses revealed that each parent harbored one of the two *ATM* mutations described above. The patient accordingly carried both germline mutations in a compound heterozygous constellation, resulting in two dysfunctional copies of the *ATM* gene. Consequently, the patient was diagnosed with ataxia teleangiectasia. Although an increased frequency of ATM-associated cancers was not apparent from the family history, the parents received genetic counseling as heterozygous carriers of a pathogenic *ATM* variant have a significantly increased risk of developing cancer [17].

Following clinical improvement of the patient and informed parent consent, the oncological treatment was continued with a single dose of rituximab and a dose-reduced AAZ1 chemotherapy cycle in line with the NHL-BFM 2012 registry (methotrexate reduced by 80%, ifosfamide reduced by 33%, cytarabine and etoposide both reduced by 50%). Additionally, the patient received three intrathecal doses of cytarabine and prednisolone. Methotrexate was not intrathecally administered due to the neurological impairment. Despite the dose adjustments, the patient suffered from prolonged bone marrow aplasia and concomitantly experienced a grade 4 oral mucositis, a subdural hematoma in the area of the ventriculoperitoneal shunt and the recurrence of pneumogenic sepsis with a previously isolated multiresistant *Pseudomonas aeruginosa*, ultimately leading to cardiac arrest while under intensive care medical treatment.

## 3. Discussion and Conclusions

We report here a rare frameshift mutation in combination with a more frequently reported mutation of the *ATM* gene in the germline of a 2-year-old patient with PCNSL, an immune deficiency disorder, and severely impaired tolerance to chemotherapy. While the girl did not present with telangiectasias on the skin or the conjunctivae, growth retardation or impaired muscle coordination in her early infancy, the PCNSL, the strongly elevated treatment toxicity and the severe bacterial infection can be directly attributed to the Louis–Bar syndrome. Since PCNSL are rarely diagnosed in children, their typical presentation, optimal treatment and prognosis can only be inferred from small retrospective studies and previous case reports. In accordance with the literature, the patient presented with typical but unspecific clinical signs of pediatric PCNSL resulting from increased intracranial pressure as well as multiple cranial nerve palsies [18]. Similar to previous cases, our patient also suffered from a central diabetes insipidus and a hypopituitarism [19]. Contrast-enhanced magnetic resonance imaging morphologically displayed a heterogeneous tumor with a marked perifocal edema, as described for pediatric PCNSL [20].

Since specific treatment protocols for pediatric PCNSL do not exist, the patient was enrolled in the NHL-BFM 2012 registry. The 3-year overall survival probability was documented to be 63 ± 12% in the cohort of 17 patients, who were registered and treated according to previous NHL-BFM registries with similar treatment schemes between 1990 and 2011 [2]. Survival appears to improve with the application of high-dose methotrexate with a minimal dose of 1 g/m^2^ as well as high-dose cytarabine to achieve sufficient drug concentrations beyond the blood–brain barrier [9,21]. Treatment of patients with an underlying immunodeficiency syndrome is much more difficult. Out of the 17 patients included in the above-mentioned cohort, all five patients with an immune deficiency disorder died due to treatment-related toxicity or complications associated with the immune deficiency disorder, indicating that the treatment of pediatric patients with PCNSL and an immune deficiency disorder remains extremely challenging [2]. The role of pre-emptive allogeneic hematopoietic stem cell transplantation in patients with ataxia teleangiectasia to restore immunocompetence and prevent malignancies is under clinical investigation [16] and underlines the importance of early cancer predisposition detection programs.

Molecular parameters, which predict patient survival and may therefore help to tailor new treatment strategies for PCNSL, are not yet clearly defined. Importantly, next-generation sequencing studies of PCNSL have identified the *ATM* gene as one of eight genes that are frequently affected by nonconservative mutations [22]. The presence of mutated *ATM* correlated with poorer event-free survival [22]. While the treatment outcome for immunocompetent children with PCNSL is generally favorable, novel treatment strategies are urgently required for patients with molecular risk factors such as *ATM* mutations and, particularly, patients with underlying germline immune deficiency disorders.

## Data Availability

Not applicable.
